# Phagocytic cell death leads to enhanced release of pro-inflammatory S100A12 in familial Mediterranean fever

**DOI:** 10.1186/s40348-023-00173-3

**Published:** 2023-12-13

**Authors:** G. Varga, S. Schleifenbaum, U. Koenig, J. Waldkirch, C. Hinze, C. Kessel, W. Geluk, T. Pap, Elke Lainka, Tilmann Kallinich, D. Foell, H. Wittkowski

**Affiliations:** 1 Department of Pediatric Rheumatology and Immunology, University Children’s Hospital Muenster, Muenster, Germany; 2 Institute of Musculoskeletal Medicine (IMM), University Hospital Muenster, Muenster, Germany; 3 Department of Pediatric Pulmonology, Immunology and Critical Care Medicine, Charité – Universitätsmedizin Berlin, Deutsches Rheuma-Forschungszentrum (DRFZ), an Institute of the Leibniz Association, Berlin, Germany; 4 Children’s Hospital, Department of Pediatric Gastroenterology, Hepatology, and Transplant Medicine, University Duisburg-Essen, Essen, Germany

**Keywords:** FMF, Neutrophils, ROS (reactive oxygen species), Gasdermin D (GSDMD)

## Abstract

**Background:**

Familial Mediterranean fever (FMF) is a prototypical autoinflammatory syndrome associated with phagocytic cell activation. Pyrin mutations are the genetic basis of this disease, and its expression has been shown in monocytes, granulocytes, dendritic cells, and synovial fibroblasts. Pyrin functions as a cytosolic pattern recognition receptor and forms a distinct pyrin inflammasome. The phagocyte-specific protein S100A12 is predominantly expressed in granulocytes and belongs to the group of damage associated molecular patterns (DAMP). S100A12 can be detected at massively elevated levels in the serum of FMF patients, even in clinically inactive disease. Whether this is crucial for FMF pathogenesis is as yet unknown, and we therefore investigated the mechanisms of S100A12 release from granulocytes of FMF patients presenting clinically inactive.

**Results:**

We demonstrate that FMF neutrophils from patients in clinical inactive disease possess an intrinsic activity leading to cell death even in exogenously unstimulated neutrophils. Cell death resembles NETosis and is dependent on ROS and pore forming protein gasdermin D (GSDMD), as inhibitors for both are capable of completely block cell death and S100A12 release. When pyrin-activator TcdA (*Clostridium difficile* toxin A) is used to stimulate, neutrophilic cell death and S100A12 release are significantly enhanced in neutrophils from FMF patients compared to neutrophils from HC.

**Conclusions:**

We are able to demonstrate that activation threshold of neutrophils from inactive FMF patients is decreased, most likely by pre-activated pyrin. FMF neutrophils present with intrinsically higher ROS production, when cultured ex vivo. This higher baseline ROS activity leads to increased GSDMD cleavage and subsequent release of, e.g., S100A12, and to increased cell death with features of NETosis and pyroptosis. We show for the first time that cell death pathways in neutrophils of inactive FMF patients are easily triggered and lead to ROS- and GSDMD-dependent activation mechanisms and possibly pathology. This could be therapeutically addressed by blocking ROS or GSDMD cleavage to decrease inflammatory outbreaks when becoming highly active.

**Supplementary Information:**

The online version contains supplementary material available at 10.1186/s40348-023-00173-3.

## Introduction

Familial Mediterranean fever (FMF) is a prototypical autoinflammatory syndrome associated with phagocytic cell activation. FMF is traditionally seen as an autosomal-recessively inherited disorder caused by mutations in the MEFV gene located on chromosome 16p that encodes a 781 amino acid protein known as pyrin (or marenostrin) ([1] International FMF Consortium, Cell, 1997 [2]; French FMF Consortium, Nat Gen, 1997). Pyrin mutations are the genetic basis of this disease, which is clinically characterized by self-limited episodes of fever and inflammation and can trigger amyloidosis as a long-term consequence, leading to organ damage. Expression of pyrin has been described in monocytes, granulocytes, dendritic cells, and synovial fibroblasts [3]. The NH2-terminal pyrin domain (PYD) can interact with apoptosis associated speck-like protein (ASC), a protein complex involved in IL-1β activation after intracellular sensing of pathogens and danger signals, leading to the formation of a distinct pyrin inflammasome [4, 5]. Pyrin functions here as a cytosolic pattern recognition receptor and triggers the formation of a capase-1 inflammasome in response to bacterial toxins [6].

The phagocyte-specific protein S100A12, which belongs to the group of damage associated molecular patterns (DAMP), can be detected at massively elevated levels in the serum of FMF patients [7]. This molecule exerts pro-inflammatory effects via interaction with pattern recognition receptors (PRRs), specifically Toll-like receptor 4 (TLR-4) [8], at concentrations that are found in FMF patients in vivo during active disease. Co-localization with the cytoskeleton and a Golgi-independent but tubulin-dependent release has been described for phagocyte-specific S100 proteins [9, 10]. This so-called alternative secretory pathway is also involved in IL-1α/β and IL-18 secretion; however, the exact mechanisms of cellular release of S100A12 are still elusive.

Recently, we have shown that FMF patients demonstrate a gene-dose effect of the pyrin mutations based on S100A12 and IL-18 serum levels [11]. Furthermore, ex vivo cultured granulocytes from FMF patients exhibit a unique phenotype with spontaneous release of high levels of IL-18, S100A12, MPO (myeloperoxidase), caspase-1, and proteinase [12], as well as activation quantified by spontaneous shedding of CD62L from the cell surface. Neutrophil activation appears to be independent of IL-1 activation and exhibits a gene-dose effect that may be responsible for the genotype-dependent phenotypes in FMF [12].

In this context, it is noteworthy that FMF patients with inactive disease—i.e., without clinical signs of disease and without elevated classical inflammatory markers (CRP and SAA)—have consistently elevated S100A12 levels [11]. Even heterozygous MEFV mutation carriers appear to have elevated S100A12 levels compared with healthy controls [13]. Whether this biochemical pattern is crucial for FMF pathogenesis, especially in the long term with respect to developing amyloidosis, is as yet unknown and should be monitored in these patients. We therefore investigated the mechanisms of S100A12 release from granulocytes of FMF patients with inactive disease.

## Patients and methods

### Patients and patient material

Patients presenting to our outpatient clinic (FMF patients and healthy controls, patient details see Table 1) routinely had clinical disease activity and parameters of inflammation (S100A8/A9, CRP, SAA, ESR, leukocytes, neutrophils) measured and retrospectively analyzed from patient charts (ethical approval by University Muenster, Ref.: 2022-703-f-S). Inflammatory diseases were excluded in the group of healthy controls. Median age of these controls was 8.8 years (4.5–16.1). Biosamples were prospectively collected from patients presenting to our outpatient clinic (FMF patients and chronic granulomatous disease (CGD) patients, patient details see Table 2) and processed as described below (ethical approval by University Muenster, Ref.: 2015-670-f-S). FMF patients that donated blood for the experiments involving granulocytes all were homozygous (see Table 2). Additionally FMF patients enrolled in the German autoinflammatory registry AID-Net were analyzed for clinical activity and S100 protein levels (ethical approval by University Muenster, Ref.: 2009-031-f-S) [11, 14].

**Table 1:** Patient demographics of the FMF cohorts and healthy controls

	Patient cohort Muenster		FMF cohort AID-Net	
	FMF homozygous	Healthy controls	Homozygous	Heterozygous
**Gender (female/male)**	9/9	10/9	21/18	8/11
**Mutations**	M694V/M694V	n/a	M694V/M694V	M694V
**Age in years at diagnosis (median, 25th–75th percentile)**	4.9 (3.4–10.9)	n/a	5.3 (3.2–7.6)	5.6 (3.6–12.0)
**Medication**	Colchicine	n/a	Colchicine	Colchicine
**CRP (mg/dl)**	<0.5	<0.5	<0.5	<0.5
**SAA (mg/l)**	9.9 (6.5–12.2) 8 from 18 neg.	5.7 (5.2–7.6) 16 from 19 neg.	3 (2–5)	2 (1–4)
**ESR (mm/h)**	9 (6–14)	5 (3–6)	12 (8–15)	8 (5–13)
**Leukocytes (per nl)**	7.7 (5.8–8.5)	6.5 (5.5–7.7)	7.2 (6.2–8.65)	6.850 (5.1–9.6)
**S10012 (ng/ml)**	n/a	n/a	170 (80–280)	81 (43–101)
**S100A8/A9 (ng/ml)**	15,730 (8975–18,730)	2040 (1400–3820)	11,225 (3896–28,627)	3938 (1650–6703)

Values are given as median and 25th–75th percentile

**Table 2:** Patient demographics of the FMF cohort, CGD patients, and healthy controls (HC) serving for granulocyte isolation and analyses

	FMF patients	CGD patients	Healthy controls
**Gender (female/male)**	8/7	2/3	4/6
**Mutations**	11× M694V/M694V 2× M694I/M694I 1× V726A/M680I 1× M680I/M694V	3× CYBB heterozygous, 2× NCF4 homozygous	n/a
**Age in years**	11 (6–13)	17.5 (7.5–18)	39 (29.3–48)
**Medication**	13× colchicine, 2× colchicine + canakinumab	Antibiotic and antimycotic prophylaxis	n/a
**CRP (mg/dl)**	0.2 (0.2–1)	<0.5	n/a
**SAA (mg/l)**	4.2 (2–38.7)	n/a	n/a
**ESR (mm/h)**	7 (5–15)	n/a	n/a
**Leukocytes (per nl)**	6.09 (5.38–8.34)	6.91	n/a
**S10012 (ng/ml)**	56 (23–1108)	56 (32–71)	n/a
**S100A8/A9 (ng/ml)**	3380 (2320–43,480)	1540	n/a

Values are given as median and 25th–75th percentile

### Isolation of primary neutrophils and subsequent stimulation and inhibition

Neutrophils were isolated from whole blood (EDTA) using MACSxpress Whole Blood Neutrophil Isolation Kit (Miltenyi Biotec, Bergisch-Gladbach, Germany) according to the manufacturer’s instructions. Purity of neutrophils was controlled by FACS staining of CD66 on isolated cells and was routinely 95% or above.

Neutrophils were either left untreated or were stimulated with PMA (100 nM, Sigma-Aldrich, Taufkirchen, Germany), with MSU crystals (200 μg/ml, InvivoGen, San Diego, CA, USA), or with *Clostridium difficile* toxin A (TcdA, 0.5 μg/ml, Sigma-Aldrich, Taufkirchen, Germany) for the indicated times and subsequently analyzed. When inhibitors were used, neutrophils were pre-incubated for 1 h before introducing neutrophils into subsequent assays or before further stimulation. ROS inhibitor DPI (diphenyleneiodonium chloride, used at 10 μM) and inhibitor of gasdermin D pore formation C23 (disulfiram—BMS-986165, used at 30 μM) were purchased from Selleckchem.com via Biozol, München, Germany. Bafilomycin A1 (1 μM) was from InvivoGen, Toulouse, France. IL-1β (5 ng/ml) was purchased from PeproTech, Hamburg, Germany. Anti-IL-1β antibody canakinumab (10 μg/ml) was from Novartis and IL-1RA anakinra (IL-1 receptor antagonist, 200 ng/ml) was from Sobi, Germany. Anti-CD66b antibody (clone g10F5) was from BioLegend, San Diego, USA. For immunoblots, anti-SQSTM1 1:2000 (Enzo, PW9860), rabbit polyclonal anti-LC3 1:2000 (GeneTex, GTX82986), anti-β-actin, and anti-GAPDH from Cell Signaling Technology, Leiden, Netherlands, were used. Immunoblots were performed as described in Koenig et al. [15].

### Cell death assay (Sytox™Green)

We performed cell death assays using SytoxGreen, a fluorescent dye that is naturally intercalating DNA, but only accessing DNA when the nuclear membrane is disintegrated.

For quantification of cell death, either 1 × 10^6^ cells/ml or 5 × 10^6^ cells/ml were plated in a 96-well plate (2 × 10^5^/well or 1 × 10^6^/well, respectively). Cells were incubated in the presence of 1.5 μM Sytox Green (Sytox™Green, ThermoFisher Scientific, Dreieich, Germany) and subsequently stimulated with the indicated activators and inhibitors. Cells were incubated for 5 h and fluorescence at 523 nm was assessed every hour using a fluorescence reader (TECAN infinite M200 pro, Tecan, Crailsheim, Germany). Supernatants were stored for S100A12 and LDH measurement.

### S100A8/A9 and S100A12 ELISA

S100A8/A9 levels were measured with Bühlmann MRP8/14 Calprotectin ELISA (Bühlmann Laboratories AG) according to manufacturer’s instructions. S100A12 in supernatants and serum was analyzed using an in-house ELISA (normal level < 150 ng/ml) as previously reported [16].

### LDH measurement

LDH measurement was done from supernatants of cells using the Pierce LDH Cytotoxicity Assay Kit (ThermoFisher Scientific, Dreieich, Germany) according to the manufacturer’s instructions.

### ROS (reactive oxygen species) measurement in flow cytometer

Ten microliters of DHR (dihydrorhodamine 123, ThermoFisher Scientific, Dreieich, Germany) was added to the cells 30 min prior to the measurement. DHR is passively transported through membranes and upon oxidation, e.g., by ROS, it becomes fluorescent and can be measured by flow cytometry (488 nm).

### Fluorescence microscopy

For SytoxGreen fluorescence, 0.5 × 10^6^/ml neutrophils were seeded in Lab-Tek chambers and then were either left untreated or were stimulated as indicated. After 4 h, supernatant was removed and NETs were then stained with 2.5 μM SytoxGreen. Stained NETs were analyzed using a fluorescence microscope at a wavelength of 488 nm emission filter with magnification 100× and 200× (Zeiss AxioVert, Zeiss, Jena, Germany).

For detection of NE (neutrophilic elastase) and histone H1 within NETs, Lab-Tek chambers were fixed with 1% paraformaldehyde for 15 min, then washed with PBS and blocked with Fc-blocking reagent. Subsequently NETs were incubated with either isotype control antibody (AF488- and AF647-conjugated) or anti-NE antibody (1 μg AF488-conjugated; Biozol, Eching, Germany) for 30 min and 1 μg anti-histone H1-AF647 antibody was used (Biozol, Eching, Germany). After 2× washing, mounting medium was added to the chambers. Glass cover slips were used on top of the mounting medium. Fluorescence images were acquired using a fluorescence microscope from Zeiss, Jena, Germany (AxioVert with Apotome). Lenses with 100× and 400× magnification were used.

### Statistical analysis

Data were analyzed with GraphPad Prism software (version 9.0 for Mac OS X, GraphPad Software, La Jolla, CA, USA), and tests applied as indicated in figure legends. Significance of differences in levels of SytoxGreen, LDH, and S100A12 content were analyzed by Brown-Forsythe and Welch’s ANOVA test followed by Dunn’s multi-comparison test. ∗*p* < 0.05, ∗∗*p* < 0.01, ∗∗∗∗*p* < 0.0001, and *p* ≤ 0.05 were considered statistically significant.

The reference values for normal controls for the “Patient cohort Muenster” in Table 1 and for patients in Table 2 are for CRP < 0.5 mg/dl and for SAA < 6.4 mg/l.

## Results

### Clinical monitoring of FMF patients confirms S100 protein release during inactive disease

**Fig. 1:**
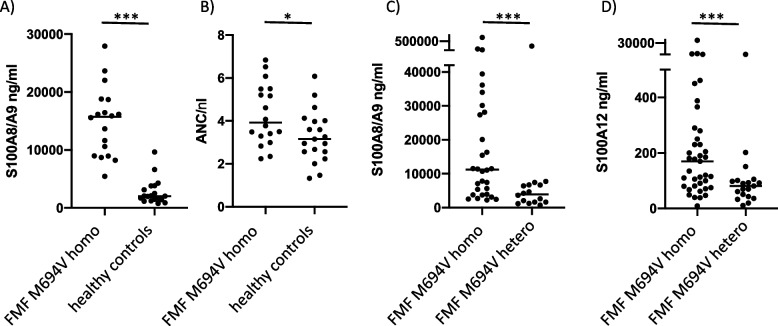
Elevated S100A12 levels in FMF patients despite inactive disease. Clinically inactive (CRP negative) FMF patients from our outpatient clinic (Table 1) were measured routinely for S100A8/A9 (**A**) and neutrophil count (**B**), showing significantly elevated levels compared to healthy controls. In a homogeneous FMF cohort (Table 1) from the AID-Net biorepository, S100A8/A9 (**C**) and S100A12 (**D**) levels were measured in homozygous and heterozygous FMF patients with the M694V genotype indicating a gene-dose effect with higher levels in homozygous patients (*p < 0.05, ***p < 0.001)

FMF patients routinely presenting to our outpatient clinic are regularly monitored for subclinical disease activity via measurement of classical inflammatory markers (CRP, SAA, and ESR) and additionally with the routinely available marker S100A8/A9. During long-term follow-up, we observed compared to healthy controls significantly elevated S100A8/A9 levels (7.7 times increase) in FMF patients with the homozygous and most serious M694V-genotype that do not have any clinical disease activity and furthermore have no increased CRP levels. Increased S100 protein levels corresponded to increased neutrophil counts (Table 1 and Fig. 1A and B).

As S100A12 levels are not available in clinical routine, and to compare with the closely related likewise phagocyte-specific S100 proteins S100A8/A9, we measured both levels in a homogeneous FMF cohort from our AID-Net biorepository. Expectedly both S100 proteins correlated highly significant (*r* = 0.82; *p* < 0.0001) and reproduced elevated levels in M649V homozygous FMF patients during inactive disease, and a gene-dose effect with significantly lower levels in heterozygous M694V FMF patients (Fig. 1C and D).

These results consistently confirmed former data from our group in homogeneous FMF cohorts, showing significantly increased S100A12 levels according to genotype and questioning the mechanism, why these patients do constantly release S100A12 despite controlled disease under colchicine treatment. We therefore investigated ex vivo granulocytes as the main source of S100A12 and analyzed cell death mechanisms as possible initiators of S100A12 release.

### Cell death induced in neutrophils from FMF patients with inactive disease

In order to analyze patient-derived neutrophilic granulocytes, we isolated neutrophils from whole blood samples as described in methods. We explicitly examined neutrophils from FMF patients in clinical remission under colchicine treatment. Patient characteristics are summarized in Table 2.

**Fig. 2:**
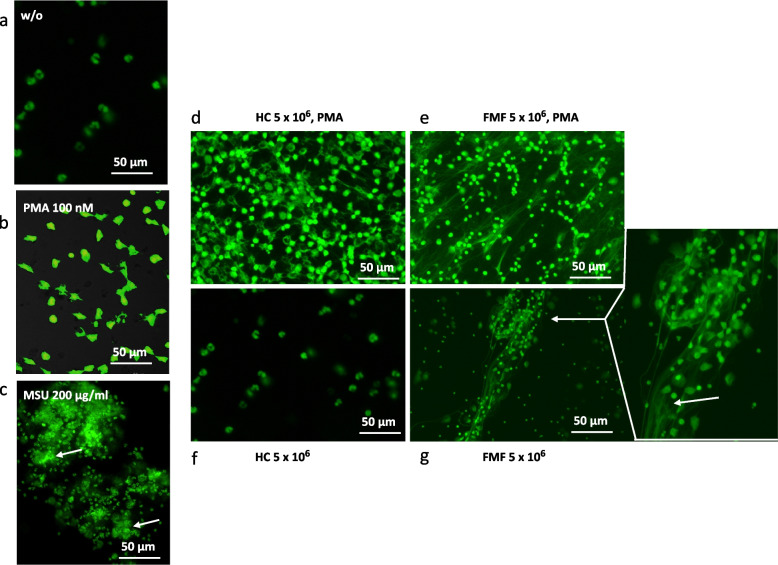
Imaging cell death in FMF neutrophils. Neutrophils from peripheral blood were isolated as described and were stained with 2.5 μM SytoxGreen. 1 × 10^6^/ml neutrophils were either left untreated or were stimulated (**a**) with PMA (**b**, 100 nM) or MSU crystals (**c**, 200 μg/ml) for 4 h. Then, photograph of cells was taken from image of fluorescence microscopy at 488 nm, scale bar 50 μm is shown (**a**–**c**). In the next experiment, 5 × 10^6^/ml neutrophils were either stimulated with PMA or left untreated for 4 h. Then cells were analyzed by fluorescence microscopy and photograph was taken at wavelength of 488 nm, magnification, scale bar of 100 μm is shown (**d**–**g**). Representative images are shown and scale bars are indicating cell size. All FMF biosamples were from homozygous patients

In FMF, it was shown that during active disease neutrophils of patients undergo NETosis that in turn regulated IL-1β-mediated inflammation [17, 18]. NETosis could be visualized by fluorescence microscopy when SytoxGreen intercalated with extruded DNA and fluorescence accumulated. This type of cell death could also be induced by stimulation of neutrophils with PMA or MSU (monosodium ureate) crystals that are built in vivo, e.g., in gout [19]. To verify, we labeled neutrophils from HC (healthy control) with SytoxGreen (Fig. 2a) and stimulated with either PMA (Fig. 2b) or MSU crystals (Fig. 2c) over a period of 4 h. As one can appreciate in Fig. 2b and c, fluorescence condensed in extruded structures upon PMA or MSU stimulation. To ensure that cell death observed with SytoxGreen includes disintegration of the nucleus, we positively stained some of the samples with antibodies against histone H1 and neutrophilic elastase (NE) suggesting that NETosis had occurred (data not shown). Stimulation of either HC or FMF neutrophils with PMA (4 h) resulted in similar phenotypes (Fig. 2d and e), while untreated neutrophils (4-h culture) differed largely. FMF neutrophils (Fig. 2g) accumulated much more SytoxGreen when compared to HC neutrophils (Fig. 2f), where fluorescence was only occasionally seen, and cells seem to be intact morphologically. However, the morphology of FMF neutrophils upon cell death resembles what has been described as “aggregated” NETs (see arrows in Fig. 2c and enlargement in h), e.g., in neutrophils from gout patients [19]. In their study, Schauer et al. demonstrated that aggregated NETS can be induced by ureate crystals in gout and occurred with rising neutrophilic cell numbers and cell densities [19].

### FMF neutrophils spontaneously underwent cell death and released S100A12

**Fig. 3:**
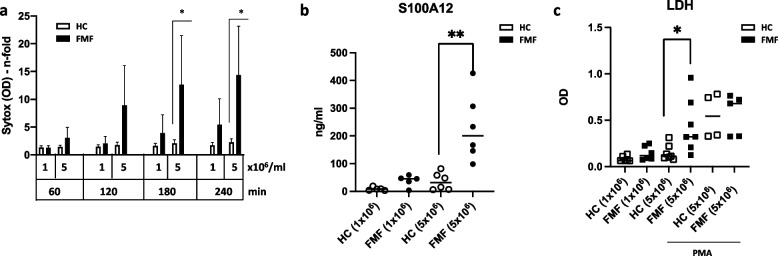
FMF neutrophils spontaneously undergo cell death and release S100A12. Neutrophils from HC (healthy control) and FMF patients were isolated as described and either seeded at 1 × 10^6^/ml or 5 × 10^6^/ml in microtiter plates after staining with SytoxGreen (2.5 μM). Cell death was quantified as amount of fluorescence at 523 nm over time (up to 4 h) (**a**). **b** Supernatants of 4-h culture were measured for S100A12 content using ELISA. **c** Supernatants of **a** (4-h culture) were measured for LDH content to independently quantify cell death. All FMF biosamples were from homozygous patients. All experiments were done at least 4 times (*n* = 4–7) and statistical significance was calculated Brown-Forsythe and Welch’s ANOVA test followed by Dunn’s multi-comparison test (* = *p* > 0.05, ** = 0.005, *** = *p* > 0.0005)

In order to verify fluorescence microscopy, we analyzed SytoxGreen intensity by quantification using a fluorescence ELISA reader. Interestingly, when cell density was 1 × 10^6^/ml, there was no significant difference in cell death when HC were compared to neutrophils from FMF patients, although there was a tendency over time that FMF neutrophils underwent stronger cell death when compared to HC (Fig. 3a, 1 × 10^6^ cells/ml). However, when cell density of 5 × 10^6^ cells/ml was used, this difference became significant over time (Fig. 3a, 180 min, 240 min). Even more interesting, this difference was also reflected when S100A12 was measured in the SN of neutrophil culture (Fig. 3b). S100A12 is one of the most prominently expressed proteins in neutrophils and the release corresponded with the amount of cell death (Fig. 3a and b). Clinically, S100A12 concentration in the serum of active FMF patients can be raised to levels significantly higher than in other fever syndromes [7]. Here, we show that S100A12 in supernatants of FMF neutrophils was significantly higher compared to HC (Fig. 3b). This confirmed our own data that were already shown by us in a similar setting [11]. Simultaneously, we measured LDH from the SN of tested neutrophils, and also here, cell death as reflected by LDH was significantly higher in neutrophil SN from FMF patients as compared to neutrophils from HC (Fig. 3c). When stimulated with PMA (Fig. 3c), the amount of LDH in the SN of HC and FMF neutrophils were very comparable suggesting that differences in unstimulated cells (HC vs. FMF) are intrinsic to FMF neutrophils.

Next, we were interested in the intrinsic mechanisms that drive spontaneous cell death of neutrophils from FMF patients. Skendros et al. [18] described an autophagy-driven and IL-1β-mediated mechanism to induce neutrophil extracellular traps in FMF neutrophils from active disease with fever attacks. In order to verify this pathway in our system using neutrophils from patients with inactive disease, we used bafilomycin A1 as an inhibitor of autophagic processing and tested neutrophils in our SytoxGreen assay (Suppl. Fig. 1). We could not find an influence of bafilomycin A1 on cell death of neutrophils (Suppl. Fig. 1a), despite bafilomycin A1 was able to block autophagy leading to an accumulation of processed MAP1LC3B (LC3-II) and SQSTM1 (p62) in HeLa cells (Suppl. Fig. 1b). Taken together, it seems that autophagy was not involved in cell death seen in our system using neutrophils from inactive FMF patients.

### Spontaneous cell death of FMF neutrophils was mediated by ROS

**Fig. 4:**
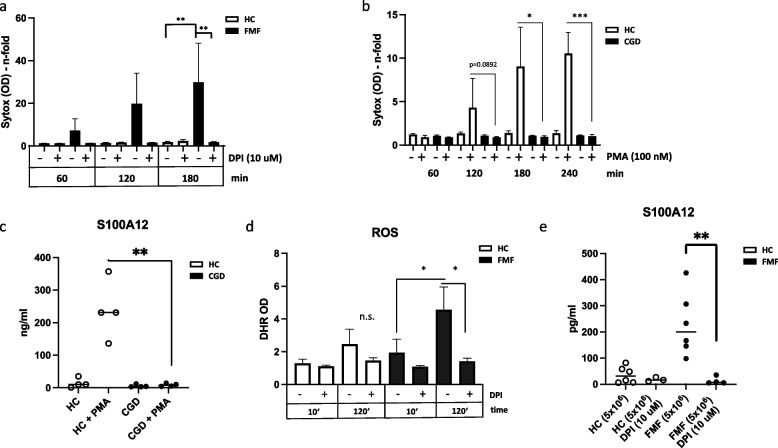
ROS pathway is driving force of cell death and directly involved in FMF. **a** Neutrophils (5 × 10^6^/ml) from HC (healthy control) and FMF patients were stained with SytoxGreen and incubated for up to 180 min with or without ROS inhibitor DPI (10 μM). Cell death assay as indicated by OD of SytoxGreen was measured for the indicated times (*n* = 3). **b** Neutrophils of HC and CGD (chronic granulomatous disease) patients (5 × 10^6^/ml) were stained with SytoxGreen and either left untreated or were stimulated with PMA for up to 4 h. OD of SytoxGreen was measured at the indicated times. Mean and SD of 4 independent experiments is shown. **c** Supernatants of **b** (after 4-h culture) were measured for content of S100A12 by ELISA (*n* = 4). Neutrophils (5 × 10^6^/ml) were incubated up to 120 min harvested and directly stained with dihydrorhodamine (DHR) to visualize ROS directly in flow cytometry (**d**, *n* = 6). Supernatants of cell cultures from **a** (120 min) were measured for S100A12 protein content using an ELISA. Data are mean and SD of 3–6 independent experiments (**e**). All FMF biosamples were from homozygous patients. Statistical significance was calculated using Brown-Forsythe and Welch’s ANOVA test followed by Dunn’s multi-comparison test (* = *p* > 0.05, ** = 0.005, *** = *p* > 0.0005)

Reactive oxygen species (ROS) have been demonstrated to be the master regulator of netotic and pyroptotic cell death [20, 21]. We therefore used diphenyleneiodonium (DPI), an inhibitor of intracellular NADPH oxidase [22], to interrupt ROS production within the neutrophils. As shown in cell death assay in Fig. 4a, spontaneous cell death of FMF neutrophils was completely and significantly blunted after 3 h of culture, indicating ROS as major inducer of cell death in FMF neutrophils. Stimulation of HC neutrophils with phorbol ester (PMA, open bars) massively induced cell death after 3 and 4 h of culture as depicted in Fig. 4b (white columns). Even more emphasis was brought to this mechanism when neutrophils from patients with chronic granulomatous disease (CGD) were tested that did not express functional NADPH oxidase in neutrophils because of a genetical disorder, and thus, their neutrophils were incapable to produce ROS [23]. These neutrophils did not undergo ROS-mediated cell death even when pushed with PMA (Fig. 4b, black columns). Even more, this was also reflected by S100A12 release and content in the supernatant of HC and CGD neutrophils (Fig. 4c).

### Intrinsic ROS accumulated in FMF neutrophils to confer ROS-dependent cell death and S100A12 release

At a density of 5 × 10^6^ neutrophils/ml, intrinsic cell activity leads to ROS-dependent cell death over time (Figs. 3a and 4a). To verify the involvement of ROS, we directly measured intracellular ROS content using dihydrorhodamine (DHR) in flow cytometry. As shown in Fig. 4d, in FMF neutrophils ROS accumulated over a period of 2 h to display significant more ROS compared to HC neutrophils and compared to the same cells at 10 min of culture. ROS inhibitor DPI could significantly block accumulation of ROS content in 2 h cultured FMF neutrophils (Fig. 4a, 2 right bars in black). Hence, basal intrinsic activity in FMF neutrophils leads to accumulation of intracellular ROS over time and subsequently to cell death. Interestingly, S100A12 released by FMF neutrophils into the supernatant was also significantly blocked by inhibition of ROS using DPI (Fig. 4e), suggesting that externalization of S100A12 coincided with cell death and disintegration of the plasma membrane.

### Pyrin inflammasome was involved in S100A12 externalization and cell death of FMF neutrophils

**Fig. 5:**
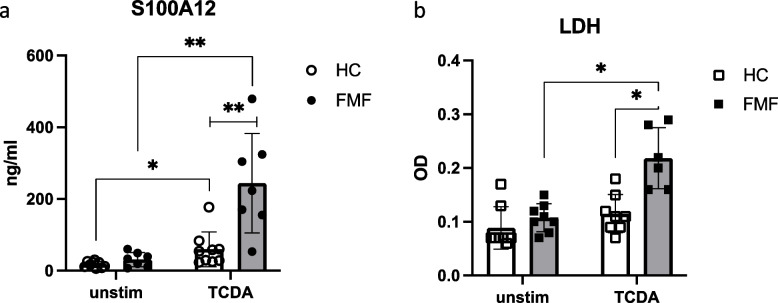
Pyrin is involved in mediating cell death. Neutrophils (5 × 10^6^/ml) from HC (healthy control) and FMF patients were stained with SytoxGreen and incubated for 4 h without or with TCDA (0.5 mg/ml). Supernatants were harvested and measured for S100A12 protein content by ELISA (**a**) and for LDH using the LDH assay kit according to the manufacturer’s protocol (**b**). Data are mean and SD of 6–8 independent individuals (**b**). All FMF biosamples were from homozygous patients. Statistical significance was calculated Brown-Forsythe and Welch’s ANOVA test followed by Dunn’s multi-comparison test (* = *p* > 0.05, ** = 0.005, *** = *p* > 0.0005)

Among others toxin of *Clostridium difficile* A (TcdA) was known to be able to activate the pyrin inflammasome [24]. To further characterize the contribution of pyrin inflammasome to cell death and S100A12 externalization, we stimulated FMF and HC neutrophils with TcdA and measured S100A12 from the supernatant (Fig. 5a). Already in HC neutrophils (Fig. 5a, white circles), stimulation with TcdA leads to significantly increased S100A12 in the supernatant of the cells. When looking at FMF neutrophils, this effect was strongly enhanced and TcdA-induced S100A12 release into the supernatant was significantly increased (Fig. 5a, black circles), suggesting that in FMF neutrophils pyrin inflammasome was already active, but could be pushed even further by pyrin activator TcdA. This effect was reflected by cell death as measured by LDH in the same supernatants (Fig. 5b), further emphasizing externalization of S100A12 presumably by disintegrated cell membrane during cell death.

### Intrinsic activity in FMF neutrophils leads to gasdermin D (GSDMD)-dependent cell death and paves the way for extracellular S100A12

**Fig. 6:**
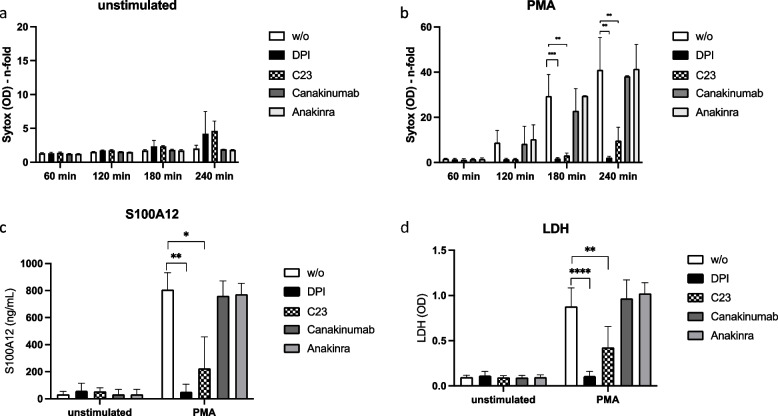
ROS-mediated cell death and S100A12 release depend on gasdermin D (GSDMD). Healthy control neutrophils (5 × 10^6^/ml) were stained with SytoxGreen and incubated for 4 h **a** without or **b** with PMA (100 nM) stimulation. Where indicated, inhibitors DPI (10 μM), C23 (30 μM), canakinumab (10 μg/ml), and anakinra (200 ng/ml) were used 1 h prior to culture. Cell death was quantified as amount of fluorescence at 523 nm. Supernatants were harvested and measured for S100A12 protein content by ELISA (**c**) and for LDH using the LDH assay kit according to the manufacturer’s protocol (**d**). Data are mean and SD of 3–8 independent individuals (**b**). Statistical significance was calculated Brown-Forsythe and Welch’s ANOVA test followed by Dunn’s multi-comparison test (* = *p* > 0.05, ** = 0.005, *** = *p* > 0.0005)

Sollberger [25] described a vital role for pore-forming protein gasdermin D (GSDMD) in the generation of neutrophil extracellular traps (NETs). Furthermore, Kanneganti et al. [26] could demonstrate that GSDMD was critical in autoinflammation in a mouse model of FMF, and Semino et al. [27] demonstrated the importance of GSDMD as one way to release IL-1β that contributes to autoinflammation in FMF. We therefore asked whether GSDMD was involved in cell death intrinsically induced in neutrophils from FMF patients in remission. Furthermore, we were interested to examine the contribution of IL-1β in this process. The results are shown in Fig. 6. We used inhibitor of GSDMD pore formation, disulfiram (C23), and IL-1RA (anakinra) as well as anti-IL-1β antibody (canakinumab) to either inhibit GSDMD-mediated effects and also IL-1-mediated effects during cell culture.

Unstimulated HC neutrophils, regardless of inhibitor treatment, did not show any significant SytoxGreen fluorescence for up to 4 h (Fig. 6a). Vastly increased, PMA-induced cell death could be seen in Fig. 6b (white bars), and as already shown in Fig. 4, ROS inhibitor DPI completely blocked PMA-induced cell death (Fig. 6b, black bars). Interestingly, also inhibitor of GSDMD pore formation, C23, was able to significantly inhibit cell death in PMA-stimulated neutrophils (Fig. 6b, hatched bars). Neither use of anakinra nor of canakinumab had any influence on cell death induced by PMA (Fig. 6b, gray bars), suggesting that IL-1β release might be the consequence of ROS-dependent and GSDMD-dependent cell death, but not their cause. Coinciding with inhibited cell death that was also confirmed by LDH measurement (Fig. 6d), we could detect significantly diminished S100A12 release when either DPI or C23 was used prior to PMA stimulation (Fig. 6c). Also here, IL-1RA (anakinra) and anti-IL-1β (canakinumab) had no influence on S100A12 release (Fig. 6c, gray bars). In order to confirm that IL-1β release was rather consequence than cause of cell death, we additionally stimulated neutrophils from HC with recombinant IL-1β (Suppl. Fig. 2) and measured SytoxGreen, LDH, and S100A12 release. We could demonstrate that IL-1β alone was not involved in ROS- and GSDMD-dependent cell death and S100A12 release (Suppl. Fig. 2). Taken together, intrinsic ROS-driven cell death pathway also involved GSDMD-induced pore formation leading to alternative protein release, e.g., among others of pro-inflammatory S100A12.

## Discussion

In our study, we describe for the first time that cell death mechanisms like NETosis and pyroptosis can intrinsically occur in neutrophils from inactive FMF patients. ROS-mediated cell death is the canonical way described in neutrophils to lead into netotic cell death [21, 28]. Cell death pathway as detected by SytoxGreen assay and LDH strongly depended on ROS in neutrophils from inactive FMF patients. Both, SytoxGreen and LDH, could be inhibited completely using DPI, an inhibitor of intracellular NADPH oxidase activity [22]. In addition, cell death also depended on pore forming protein GSDMD that originally was described to be triggered by NLRP3 inflammasome to drive pyroptosis and to be independent of NETosis [29]. Recently, it became more and more evident that GSDMD also plays a role in other cell death pathways than pyroptosis, namely, NETosis and even apoptosis [20, 25].

Apparently, neutrophils from inactive FMF patients are readily activated by intrinsic pyrin activity that presumably lowers activation threshold for subsequent mechanisms like cell death pathways. In monocytes from FMF patients, it was demonstrated that stimulation via pyrin leads to enhanced inflammation and cytokine responses compared to monocytes from HC. However, when other inflammasomes were addressed, e.g., NLRP3 and NLRC4, the inflammatory response of the monocytes did not differ. The authors conclude that FMF-associated MEFV mutations decrease the activation threshold of the pyrin inflammasome without altering other inflammasomes [30]. So, it is conceivable that pre-activated pyrin in neutrophils from FMF patients could accumulate pyrin-inflammasome-dependent signals, e.g., with rising cell-to-cell contact that leads into overactivation and cell death. It is, however, not known to date whether these mediators are surface molecules that profit from cell-to-cell contact in rising cell densities or soluble factors that profit from short distances. Cell density seems to play a role for initiation of NETosis as demonstrated in gout-induced aggregated NETs [19]. Recently, we described neutrophils from also clinically inactive FMF patients already displaying increased activation measured by ex vivo shedding of L-selectin without any additional external stimulation, and this was following a gene-dose effect [12]. Additionally we already described the release of S100A12, IL-18, caspase-1, and MPO from these granulocytes without further stimulus and we can now add to this the probable cell death mechanism responsible for at least S100A12 release.

Apostolidou et al. [17] and Skendros et al. [18] described netotic cell death in neutrophils from FMF patients in disease attack that was dependent on autophagy and led to release and display of IL-1β to the extracellular space and the released IL-1β was able to drive inflammation. By using inhibitor of autophagy in our SytoxGreen assay, we could not detect any contribution of autophagy to the ROS-induced cell death.

Kanneganti et al. [26] demonstrated in a mouse model of FMF that gasdermin D was required for autoinflammatory pathology. Here, one major player was IL-1β that was shown to be secreted through GSDMD pores and contributed to pathology. The whole process was driven by infection of mice with *Clostridium difficile* (*C. difficile*) to induce NLRP3 inflammasome leading to pyroptosis and IL-1β release through pore forming GSDMD. In their study, they utilized macrophages as major source of Il-1β. Also Evavold et al. [31] demonstrated that GSDMD regulates IL-1β secretion in living macrophages. When we used neutrophils from inactive FMF patients, we could not detect a direct influence of IL-1β leading to auto-driven cell death, as shown by the use of anti-IL-1 antibody (canakinumab) and IL-1RA (anakinra) in our model. Neither canakinumab nor anakinra had an influence on cell death in FMF neutrophils (Sytox assay, LDH). We also used recombinant IL-1β to induce cell death and S100A12 release from neutrophils but were unable to detect cell death and protein release (Supp. Fig. 2).

However, release of DAMP S100A12 was significantly blocked when inhibitor of GSDMD cleavage and pore forming, C23 (disulfiram), was used in cell culture. This directly points to the importance of GSDMD activation and cleavage as a major mechanism for “alternative” release of cytokines and proteins into the extracellular space. We recently [11] showed that neutrophils from FMF patients already released S100A12 (and also IL-18 and Casp-1) spontaneously, presumably through the GSDMD cleavage and pore formation. In addition, we could already demonstrate that S100A12 acts as a pro-inflammatory DAMP on human cells [32]. Even more, Jorch et al. [33] demonstrated the importance of S100A8/9 release through GSDMD pores for FMF pathology in a mouse model of FMF and in cell lines. We expand this knowledge by adding S1000A12 to the list of GSDMD pore-regulated proteins in neutrophils. Additionally, S100A12 release from neutrophils is also dependent on ROS, as neutrophils from CGD patients, which do not build active ROS intrinsically, do not release S100A12, not even when stimulated with PMA (Fig. 4). Semino et al. [27] demonstrated that blocking ROS production also prevents GSDMD cleavage claiming the importance of oxidative stress in GSDMD-mediated secretion. We confirm here this finding by examining S100A12 release from human neutrophils. When we stimulate neutrophils from HC with PMA, S100A12 is released. This can be blocked by both, ROS inhibitor and by GSDMD inhibitor, suggesting that ROS can also drive GSDMD cleavage. Sollberger [25] already found GSDMD to play a vital role in the generation of extracellular traps. Especially, when we induce NETosis by using PMA, the role of GSDMD becomes evident, because in this case GSDM inhibitor not only blocked S100A12 release but also cell death.

We used TCDA to trigger cell death and S100A12 release to demonstrate that pyrin inflammasome is involved in cell death and ROS production. We could show that TCDA induced stronger S100A12 release and cell death when neutrophils were from FMF patients, although inactive clinically, they apparently present with pre-activated pyrin inflammasome. However, TCDA was shown to also activate NLRP3 inflammasome [34, 35]. Thus, there is additional experimentation necessary to detect contribution of either pyrin and/or NLRP3 inflammasome to cell death and GSDMD activation, and subsequent protein release. Limitations of our study are certainly limited number of patients and missing age matched controls from pediatric individuals.

## Conclusion

We demonstrate that activation threshold of neutrophils from inactive FMF patients is decreased, most likely by pre-activated pyrin. FMF neutrophils present with intrinsically higher ROS production, when cultured ex vivo. This higher baseline ROS activity leads to increased GSDMD cleavage and subsequent release of, e.g., S100A12, and to increased cell death with features of NETosis and pyroptosis. We show for the first time that cell death pathways in neutrophils of inactive FMF patients are easily triggered and lead to ROS- and GSDMD-dependent activation mechanisms and pathology. This could be therapeutically addressed by blocking ROS or GSDMD cleavage to possibly decrease inflammatory outbreaks in FMF patients.

### Supplementary Information

**Additional file 1: Supplemental Figure 1.** Autophagy does not seem to be involved in spontaneous cell death. Neutrophils were isolated as described and either seeded at 5 × 10^6^/ml in a microtiter plate after staining with SytoxGreen (2.5 μM). Cell death was quantified as amount of fluorescence at 523 nm over time (up to 4 h) (**a**, *n* = 4). Where indicated, autophagy inhibitor bafilomycin (1 μM) was added to the cells. **b** HeLa cells were cultured o.n. in the presence of 0.1, 0.5, and 1.0 μM bafilomycin. Cells were harvested and western blot performed using antibodies against LC3 and p62 that accumulate when autophagy is stopped. All FMF biosamples were from homozygous patients. Western blot shown is representative of 2 independent experiments.

**Additional file 2: **Supplemental Figure 2. Contribution of IL-1β on cell death of neutrophils. Neutrophils from HC were isolated as described and seeded at 5 × 10^6^ cells/ml in a 96-well microtiter plate after staining with SytoxGreen (2.5 μM). Cells were either left untreated or were stimulated with PMA (100 nM) and IL-1b (5 ng/ml). Cell death was quantified as amount of fluorescence at 523 nm over time up to 4 h (**a**). Supernatants were harvested and measured for S100A12 protein content by ELISA (**b**) and for LDH using the LDH assay kit according to the manufacturer’s protocol (**c**). All FMF biosamples were from homozygous patients. Results are shown of *n* = 2 independent experiments.

## Data Availability

The datasets used and/or analyzed during the current study are available from the corresponding author on reasonable request.

## Abbreviations

CGD: Chronic granulomatous disease
DAMP: Danger associated molecular pattern
FMF: Familial Mediterranean fever
GSDMD: Gasdermin D
HC: Healthy control
NET: Neutrophil extracellular trap
ROS: Reactive oxygen species
TcdA: *Clostridium difficile* toxin A
