# Supplementary material for: Phagocytic cell death leads to enhanced release of pro-inflammatory S100A12 in familial Mediterranean fever
Source: Mol Cell Pediatr. 2023 Dec 13;10:19. doi: 10.1186/s40348-023-00173-3 (PMC10716088; doi:10.1186/s40348-023-00173-3)
Supplement: Supplementary file 1 — Additional file 1: Supplemental Figure 1. Autophagy does not seem to be involved in spontaneous cell death. Neutrophils were isolated as described and either seeded at 5 × 10^6^/ml in a microtiter plate after staining with SytoxGreen (2.5 μM). Cell death was quantified as amount of fluorescence at 523 nm over time (up to 4 h) (a, n = 4). Where indicated, autophagy inhibitor bafilomycin (1 μM) was added to the cells. b HeLa cells were cultured o.n. in the presence of 0.1, 0.5, and 1.0 μM bafilomycin. Cells were harvested and western blot performed using antibodies against LC3 and p62 that accumulate when autophagy is stopped. All FMF biosamples were from homozygous patients. Western blot shown is representative of 2 independent experiments. [file 40348_2023_173_MOESM1_ESM.pptx]

## Slide 1
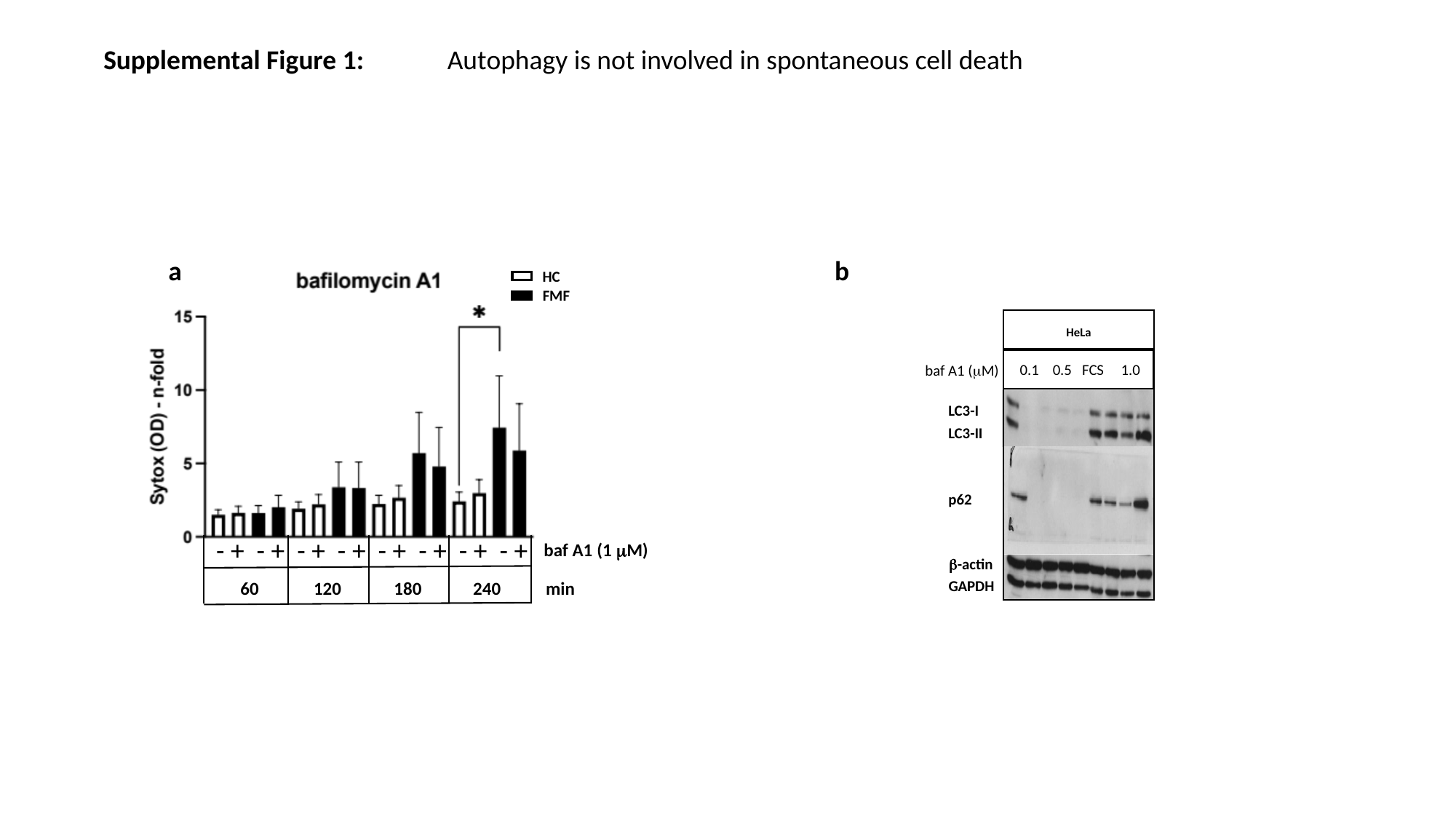

Supplemental Figure 1:
Autophagy is not involved in spontaneous cell death
a
HC
FMF
- + - + - + - + - + - + - + - +
60
120
180
240
baf A1 (1 mM)
min
b
HeLa
0.1 0.5 FCS 1.0
baf A1 (mM)
LC3-I
LC3-II
p62
b-actin
GAPDH
