# Supplementary figures and images for: Phagocytic cell death leads to enhanced release of pro-inflammatory S100A12 in familial Mediterranean fever
Source: Mol Cell Pediatr. 2023 Dec 13;10:19. doi: 10.1186/s40348-023-00173-3 (PMC10716088; doi:10.1186/s40348-023-00173-3)

## Slide 1
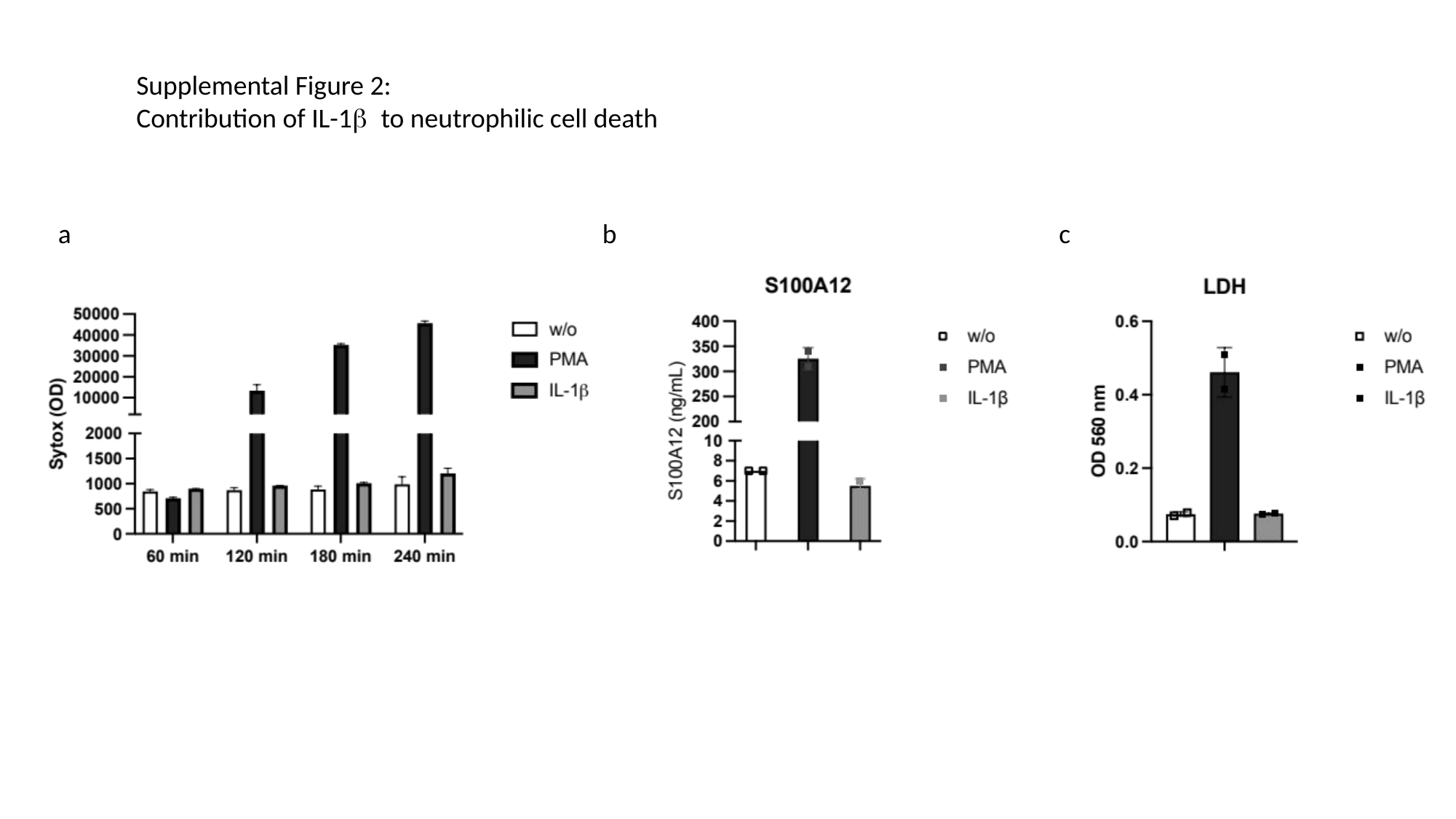

Supplemental Figure 2:
Contribution of IL-1b to neutrophilic cell death
a
b
c

Supplement: Supplementary file 2 — Additional file 2: Supplemental Figure 2. Contribution of IL-1β on cell death of neutrophils. Neutrophils from HC were isolated as described and seeded at 5 × 10^6^ cells/ml in a 96-well microtiter plate after staining with SytoxGreen (2.5 μM). Cells were either left untreated or were stimulated with PMA (100 nM) and IL-1b (5 ng/ml). Cell death was quantified as amount of fluorescence at 523 nm over time up to 4 h (a). Supernatants were harvested and measured for S100A12 protein content by ELISA (b) and for LDH using the LDH assay kit according to the manufacturer’s protocol (c). All FMF biosamples were from homozygous patients. Results are shown of n = 2 independent experiments. [file 40348_2023_173_MOESM2_ESM.pptx]
